# Practical approach to detection and management of acute kidney injury in critically ill patient

**DOI:** 10.1186/s40560-017-0251-y

**Published:** 2017-09-16

**Authors:** Vahid Mohsenin

**Affiliations:** 1 Section of Pulmonary, Critical Care and Sleep Medicine, Yale School of Medicine, New Haven, CT USA; 2 Department of Medicine, Lippard Laboratory of Clinical Investigation, Yale School of Medicine, 15 York Street, LLCI-106-E, New Haven, CT 06510 USA

**Keywords:** Acute kidney injury, Fluid volume assessment, Urine microscopy, Critical illness

## Abstract

**Background:**

Acute kidney injury (AKI) is a common complication in critically ill patients and is associated with high morbidity and mortality. This paper provides a critical review of the etiologies of AKI and a systematic approach toward its diagnosis and management with emphasis on fluid volume assessment and the use of urine biochemical profile and microscopy in identifying the nature and the site of kidney injury.

**Materials and methods:**

The search of PubMed and selection of papers had employed observational designs or randomized control trials relevant to AKI.

**Results:**

AKI is defined by the rate of rise of serum creatinine and a decline in urine output. The pathophysiology is diverse and requires a careful and systematic assessment of predisposing factors and localization of site of injury. The majority of AKIs are due to prerenal causes such as fluid volume deficit, sepsis, or renal as in acute tubular injury. The use of central venous and arterial blood pressure monitoring and inferior vena cava echocardiography complemented by urine analysis and microscopy allows assessment of fluid volume status and AKI etiology.

**Conclusions:**

Timely intervention by avoidance of fluid volume deficit and nephrotoxic agents and blood pressure support can reduce the incidence of AKI in critically ill patients.

## Background

Acute kidney injury (AKI) is a common complication among patients with critical illness in the intensive care unit (ICU). The incidence of AKI is now believed to be significantly higher than previously believed with over 50% of patients in the ICU developing AKI at some point during the course of their critical illness. Mortality among ICU patients with AKI and multi-organ failure has been reported to be more than 50% [1–3]. Those that require renal replacement therapy (RRT) mortality may be as high as 80% [4–6]. AKI is characterized by a sudden decrease in kidney function over a period of hours to days, resulting in accumulation of creatinine, urea, and other waste products. A consensus definition of AKI and formulation of the RIFLE criteria—acronym RIFLE stands for Risk, Injury, and Failure; and the two outcome classes, Loss and End-Stage Renal Disease [7, 8]—is based on the degree of rise of serum creatinine, decreased in glomerular filtration rate (GFR), and urine output. The most recent definition of AKI represents a harmonization of the previous RIFLE and Acute Kidney Injury Network (AKIN) [8] classifications with increased sensitivity for diagnosis of AKI. According to the 2012 Kidney Disease: Improving Global Outcomes (KDIGO) consensus guidelines, AKI is defined by an increase in the serum creatinine level of 0.3 mg/dL (26.5 μmol/L) or more within 48 h; a serum creatinine level that has increased by at least 1.5 times the baseline value within the previous 7 days; or a urine volume of less than 0.5 mL per kilogram of body weight per hour for 6 h. For AKI staging purposes, patients should be staged according to the criteria that give them the highest stage (Table 1) [9]. However, this definition does not offer any understanding of pathophysiological mechanism underlying AKI; it can be thought of more like acute lung injury. AKI is the consensus term for acute renal failure [10]. The term AKI meant to imply that an injury process existed before any loss of kidney function can be measured with standard laboratory tests. The main criterion used to define AKI is the rate of rise of serum creatinine. However, the degree of renal dysfunction can be underestimated due to changes in the volume of distribution, falls in creatinine generation in patients with advanced chronic kidney disease (CKD) [11], and loss of muscle mass in critically ill patients [12, 13].

**Table 1:** Definition and staging of acute kidney injury: KDIGO criteria

Stage	Serum creatinine	Urine output
1	1.5–1.9 times baseline OR ≥ 0.3 mg/dL (≥ 26.5 μmol/L) increase	< 0.5 mL/kg/h for 6–12 h
2	2.0–2.9 times baseline	< 0.5 mL/kg/h for ≥ 12 h
3	3.0 times baseline OR Increase in serum creatinine to ≥ 4.0 mg/dL (≥ 353.6 μmol/L) OR Initiation of renal replacement therapy OR, in patients < 18 years, decrease in eGFR to < 35 mL/min per 1.73 m^2^	< 0.3 mL/kg/min for ≥ 24 h OR Anuria ≥ 12 h

The time-honored classification of AKI to prerenal azotemia, intrinsic renal diseases, and post-renal urinary obstruction provides a conceptual framework by which pathophysiological mechanisms can be investigated [14]. The current evidence supports the validity of KDIGO criteria to identify groups of hospitalized patients with increased risk of death and/or need for RRT. There is accumulating evidence of long-term risk of subsequent development of cardiovascular disease or CKD, even after apparent resolution of AKI [15–17].

## Causes of AKI

The pathogenesis of AKI is complex due to multiple etiologies and risk factors (Table 2). Risk factors include increasing age, presence of heart failure, liver failure, CKD, anemia, and exposures to nephrotoxic agents including antibiotics, non-steroidal anti-inflammatory drugs (NSAIDS), and radiocontrast dyes. Infections, sepsis, shock, need for mechanical ventilation, and surgery are well recognized as high-risk settings for the development of AKI [18]. AKI is a broad clinical syndrome encompassing various etiologies, including specific kidney diseases (e.g., acute tubular necrosis, acute interstitial nephritis, acute glomerular, and vasculitis renal diseases), non-specific conditions (e.g., ischemia, toxic injury), and extrarenal pathology (e.g., prerenal azotemia and acute post-renal obstructive nephropathy).

**Table 2:** Etiologies of AKI

Prerenal AKI
• Dehydration (vomiting, diarrhea) • Bleeding or hypovolemia • Heart failure • Liver failure • Narrowing of renal arteries • Renal microangiopathy • Exposure to vasoactive drugs
Renal AKI
• Acute tubular injury/toxicity • Drugs (common in ICU) • Aminoglycosides, vancomycin • Amphotericin B, pentamidine • Contrast dyes • Bisphosphonates • Cisplatin, ifosfamide, methotrexate (crystal nephropathy) • Tumor lysis syndrome • Multiple myeloma • Rhabdomyolysis (crush injury, cocaine, heroin, ketamine, methadone, and methamphetamine) • Tissue hypoperfusion, sepsis • Acute interstitial nephritis • Vancomycin, quinolones, rifampin, sulfonamides, β-lactams (penicillin, cephalosporins), acyclovir (crystal nephropathy) • NSAIDs • Ifosfamide, carboplatin, Adriamycin, tyrosine kinase inhibitor
Post-renal AKI reflects the obstruction of the urinary system, particularly the ureters.

Prerenal AKI including sepsis accounts for 60–70% of all AKI cases in critically ill patients [1]. Renal blood flow (RBF) autoregulation through its synchronized interplay of afferent and efferent arterioles maintains a constant RBF and GFR within a wide range of mean arterial blood pressure (80–180 mmHg) [19]. A decline in the GFR reflects a reduction in blood pressure below the lower limit of the autoregulatory range. However, a significant decline in blood pressure must occur to induce a sufficient reduction in GFR with subsequent development of AKI. In the tubuloglomerular feedback (TGF) mechanism through *macula densa*, when blood pressure decreases, the vasodilators (vasodilator prostaglandins and nitric oxide) are released with afferent arteriolar vasodilation and subsequent stimulation of renin-angiotensin-aldosterone system (RAAS) [20, 21]. The opposite occurs when blood pressure increases with generation of vasoconstrictor mediators (vasoconstrictor ATP and adenosine), causing afferent arteriolar vasoconstriction while the efferent arteriole dilates to stabilize RBF and GFR. The TGF mechanism and the myogenic activity of the afferent arteriole provide an estimated 90% of the autoregulation capacity of RBF [22]. Arterial blood pressure alone cannot sustain GFR, so these important autoregulatory mechanisms involving the TGF- and RAAS-mediated compensations are needed. Risk of AKI increases when these mechanisms are inhibited through angiotensin converting enzyme inhibitors (ACEI) and NSAID [23, 24].

Sepsis and septic shock remain the most important causes of AKI in critically ill patients and account for more than 50% of cases of AKI in the ICU [3]. The common assumption has been that tissue hypoperfusion and renal ischemia are the mechanisms of AKI in sepsis and hyperdynamic septic shock. An increasing body of evidence suggests that in a significant proportion of patients, AKI can occur in the absence of overt signs of tissue hypoperfusion, suggesting that other mechanisms may be at work. Studies in animal models of sepsis [25, 26] and humans with sepsis and AKI [27, 28] have shown that as part of the systemic vasodilation observed during severe sepsis/septic shock, RBF is not decreased and even increased indicating that AKI occurs not in the setting of renal hypoperfusion. Furthermore, in septic animals and in postmortem observations in humans with sepsis-induced AKI, kidney histology is strikingly bland with focal areas of tubular injury, but minimal cell death [29, 30]. In aggregate, these observations suggest the presence of three distinct perturbations as mechanisms of AKI in sepsis: diffuse microcirculatory flow abnormalities, inflammation, and cellular bioenergetic responses to injury [31, 32].

Renal AKI develops when the primary cause of AKI is parenchymal injury, caused by drugs, toxins, and ischemia. The mechanisms underlying the renal injury process in AKI have predominantly been studied in animal models of ischemic AKI, characterized by “activation” of epithelial and possibly endothelial cells during the early initiation phase results in the upregulation of a variety of chemokines and cytokines leading up to inflammatory cell infiltration including macrophages with loss of the brush border in the proximal tubular epithelium [33]. Nephrotoxic agents have been implicated as etiologic factors in 17–26% of in-hospital AKI [3, 34]. Patient’s specific risk factors for drug nephrotoxicity are older age, female sex, hyperbilirubinemia, fluid volume depletion, and hypoalbuminemia by inducing toxic drug levels and increasing the unbound drug fraction in the serum. ACEI and angiotensin II receptor blockers could precipitate or potentiate AKI in certain situations. These include (1) bilateral renal artery stenosis or renal artery stenosis in solitary kidney, (2) volume depletion, and (3) concomitant use of NSAIDs, cyclosporine, and tacrolimus [35].

## Diagnosis

AKI is diagnosed when serum creatinine increases by ≥ 0.3 mg/dL (≥ 26.5 μmol/L) within 48 h or increase in serum creatinine to ≥ 1.5 times baseline within the previous 7 days or urine volume < 0.5 mL/kg/h for 6 h [9].

### Fluid volume assessment

The real challenge is to determine the cause of AKI. The first order is to assess the fluid volume status of the patient. This can be accomplished by estimating the fluid volume balance in the preceding days, blood pressure trend, and assessment of intravascular volume. Intravascular volume is not measured directly but can be inferred by the presence or absence of fluid responsiveness. Echocardiography examining the inferior vena cava diameter and its respiratory variation is becoming readily available in ICUs. More than 12% variation in the IVC diameter in mechanically ventilated patient with septic shock had greater than 90% positive predictive value (PPV) for fluid responsiveness implying intravascular volume deficit [36]. Central venous pressure (CVP) dynamic changes or arterial pulse pressure variation during respiratory cycle can also provide information about intravascular volume deficit [37]. A drop of ≥ 1 mmHg in CVP during spontaneous inspiration or mechanical breath had a PPV of 84% and negative predictive value of 94% for fluid responsiveness in a mixed medical and surgical ICU [38]. In arterial pulse pressure variation of > 13% during respiratory cycle, the respective values were 94% and 96% [39].

### Urinary and serum biochemical measurements

Urinary diagnostic indices can have confirmatory value (Table 3). Concentrated urine with urine-specific gravity > 1.020, BUN/Cr > 20:1, urine Na < 20 mEq/L, or low FeNa < 1.0% are consistent with fluid volume deficit or renal hypoperfusion denoting a prerenal AKI. On the other hand, rising serum creatinine with FeNa > 2.0% highly suggests renal AKI. However, some caveats should be noted with these measures as outlined in Table 3. Studies on biochemical analysis of urine using standard measurements of sodium, urea, and creatinine and calculating various indices of tubular function, FeNa, and FeUrea are not diagnostically accurate, prognostically valuable, or clinically useful in septic patients with AKI [40, 41]. In patients with oliguria, fluid challenge reversed oliguria in only one half of the patients and that neither urinary sodium, FeNa, nor FeUrea was a good predictor of renal response to fluid challenge [42]. Therefore, these parameters must always be interpreted within the clinical context and in conjunction with hemodynamic measurements.

**Table 3:** Biochemical parameters in prerenal and renal AKI

Parameters	Prerenal	Renal	Comments
Urine-specific gravity	> 1.020	1.008–1.012	In chronic kidney disease and renal AKI, urine-specific gravity is not reliable in the assessment of intravascular volume depletion due to lack of renal concentrating ability
BUN/Cr	> 20:1	10:1	In prerenal state, BUN is absorbed in proximal tubules out of proportion to GFR and serum creatinine, increasing the BUN/Cr ratio. Caveats: Steroid therapy and low muscle mass can increase the ratio and decreased protein intake can lower the ratio.
Urine sodium	< 20 mEq/L	> 20 mEq/L	>20 mEq/L in ATN and diuretic therapy
FeNa	< 1.0%	> 2.0%	Caveats: Low FeNa is seen in contrast nephropathy, rhabdomyolysis, glomerulonephritis, vasculitis, and acute tubular necrosis (ATN) in the setting of cirrhosis and congestive heart failure. High FeNa (> 2.0%) is seen in AKI (e.g., ATN) and with diuretic use even in the setting of shock and hypovolemia
FeUrea	< 35%	> 50%	Useful in the setting of diuretic use

*ATN* acute tubular necrosis, *FeNa* fractional excretion of sodium, *FeUrea* fractional excretion of urea

### Biomarkers of kidney injury

The current definition and classification of AKI tell nothing about whether the dysfunction is purely functional or completely structural—these two extremes likely do not exist. Serum creatinine is the defining indicator of AKI in KDIGO guideline. But it has been shown, serum creatinine, to be a lagging indicator of AKI development and it is easily influenced by many factors, including sex, muscle mass, and some medications.

The search for early identification of AKI has led to the development of multiple biomarkers of AKI capable of detecting kidney injury at its early stage. A series of molecules have been evaluated over the years, and significant advances have been made in the field. Several biomarkers have been shown to have predictive ability in recognizing kidney damage earlier than creatinine but have not entered mainstream use as yet. It has been proposed to measure these biomarkers as functional (e.g., serum creatinine, serum cystatin C) and damage (e.g., NGAL, neutrophil gelatinase-associated lipocalin; KIM-1, kidney injury molecule-1; TIMP2, tissue inhibitor of metalloproteinases 2; IGFBP3, insulin-like growth factor-binding protein 3) markers in combination to improve the diagnostic categorization of AKI and permit more guided interventions [43].

Cystatin C is a small protein produced by nucleated cells and eliminated by GFR. It behaves therefore similar to serum creatinine but is less dependent on muscle mass. In ICU patients, cystatin C will detect AKI 1–2 days earlier before the rise of serum creatinine. Its costly measurement limits its routine use.

Molecules such as NGAL and KIM-1 have demonstrated some capacity to detect an injury to the kidney well before the rise of serum creatinine can be observed. Measurement of serum or urine NGAL has been shown to be a good diagnostic test for AKI and prognostic indicator of RRT need and mortality in patients with shock in many studies [44–46] but not all [47]. However, routine use of NGAL in clinical setting is limited due to its increase in both serum and urine which may be related to the presence of a systemic inflammatory state including sepsis and not necessarily the development of AKI [48].

In 2013, the SAPPHIRE study was the first investigation to find and validate the two top urinary biomarkers for the prediction of AKI risk among 340 proteins in a discovery cohort. These biomarkers, urine insulin-like growth factor-binding protein 7 (IGFBP7) and tissue inhibitor of metalloproteinase-2 (TIMP-2), were then validated in > 700 critically ill adult patients [49]. A simple product of these biomarker levels (TIMP-2 × IGFBP7), expressed in (ng/mL)^2^/1000, launched as a final commercial product, known as NephroCheck. The cutoffs of > 0.3 and > 2 (ng/mL)^2^/1000 were associated with a progressively increased risk of AKI and major adverse kidney events (death, the need of RRT or persistent renal dysfunction) [49]. NephroCheck of < 0.3 (ng/mL)^2^/1000 had a negative predictive value of 96% following adjustment for the prevalence of AKI [50–52]. These kidney damage biomarkers are currently under evaluation for routine clinical use.

### Urine microscopy

**Fig. 1:**
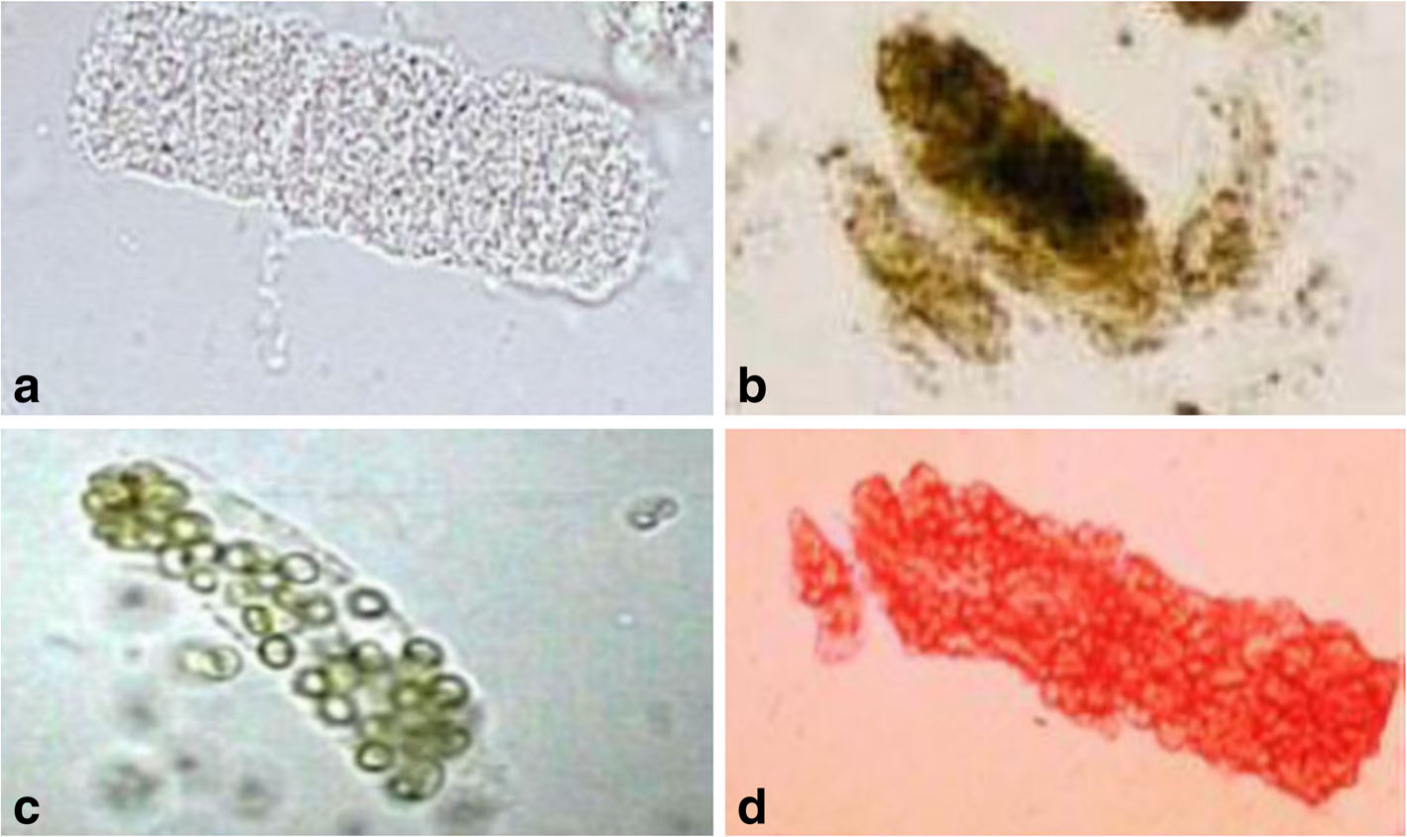
Urine microscopy for analysis of urine sediments. Renal tubular epithelial cell casts (**a**) and “Muddy” brown granular casts (**b**) suggest acute tubular injury/necrosis (ATN) as the etiology of AKI. White blood cell casts are generally seen in acute interstitial nephritis or acute pyelonephritis (**c**). Red cell cast denotes glomerular disease as in glomerulonephritis or small vessel vasculitis (**d**)

Examination of urine sediments by microscopy can provide insight into the etiology of renal AKI [53]. Urine sediment in prerenal AKI is generally bland with some hyaline casts and no cellular shedding or crystals. The presence of renal tubular epithelial cell casts (Fig. 1a) and “Muddy” brown granular casts (Fig. 1b) suggests acute tubular injury/necrosis (ATN) as the etiology of AKI. White blood cell casts are generally seen in acute interstitial nephritis or acute pyelonephritis (Fig. 1c). Red cell cast denotes glomerular disease as in glomerulonephritis or small vessel vasculitis (Fig. 1d). Urine sediments can not only help to differentiate prerenal AKI from renal AKI but can also provide insight as to the site of nephron injury.

## Management of AKI

### Fluid volume expansion

If clinical assessment points toward intravascular volume deficit, optimization of the hemodynamic status and correction of any volume deficit should have a salutary effect on kidney function and help to minimize further extension of the kidney injury [54]. Fluid resuscitation should be done while monitoring urine output, blood pressure, or CVP dynamic changes as endpoints to avoid excessive fluid administration. In a retrospective analysis of 105 ICU patients, a linear correlation was found between elevated levels of CVP and AKI incidence or its duration [55]. This is due to increased venous congestion and decreased pressure gradient across the glomerulus lowering GFR. Large multicenter studies have shown that a positive fluid balance is an important factor associated with increased 60-day mortality [56–58]. Observational data suggest that buffered crystalloids with low-chloride content may be associated with a decreased risk of AKI [59, 60]. The plausibility of this notion is that the unphysiologic chloride concentration (154 mEq/L) in normal saline can cause renal vasoconstriction, decreased glomerular filtration, and metabolic acidosis [61, 62]. However, a recent randomized controlled trial of low-chloride buffered crystalloid solution vs. normal saline showed no difference in the incidence of AKI in a heterogeneous population of patients treated in ICU [63]. As pointed out by the authors of this trial, further studies are needed to assess efficacy of buffered crystalloid solutions in higher-risk populations and to measure clinical outcomes such as mortality.

Colloid solutions such albumin can be used in patients with hypoalbuminemia in the setting of fluid volume deficit, hypotension, and AKI. Albumin infusion is also indicated in patients with liver cirrhosis with AKI in the form of hepatorenal syndrome in conjunction with vasopressors.

### Vasopressors

In septic shock with AKI norepinephrine is the vasopressor of choice with target mean arterial pressure of 65–70 mmHg. However, in patient with chronic hypertension, a higher target mean arterial pressure of 80–85 mmHg is recommended [64]. The target mean arterial blood pressure of 65–70 mmHg is also applicable in patients with hepatorenal syndrome [65, 66]. In hepatorenal syndrome, all vasoconstrictors should include cotherapy with albumin.

### Diuretics

Earlier studies have demonstrated improved outcome with aggressive use of loop diuretics in AKI despite an increase in serum creatinine [67–69]. A study demonstrated that a urinary output of at least 100 ml/h following a test dose of 1.0–1.5 mg furosemide per kilogram body weight predicted lower risk of progression to a higher stage of AKI in oliguric patients [70]. However, in a multi-center randomized controlled trial in adult patients with AKI, low-dose furosemide (0.4 mg/kg loading dose followed by a continuous infusion commenced at a dose of 0.05 mg/kg/h) did not reduce the rate of worsening of AKI, improve recovery, or reduce the need for RRT [71]. This seemingly disparate findings are likely related to the differences in pharmacokinetics and pharmacodynamics of furosemide due to the degree of impairment of creatinine clearance in patients with AKI. In this study, measured creatinine clearance was strongly associated with the amount of urinary furosemide excreted and was the only reliable predictor of the urinary output after furosemide [72]. In a recent statement, the European Society of Intensive Care Medicine recommended against loop diuretics given solely for the prevention of AKI [64]. However, in patient with AKI and hypervolemia such as in cardiorenal syndrome with decompensated heart failure, loop diuretics should be employed. Diuretics in the setting of AKI have not been shown to cause nephrotoxicity.

### Renal replacement therapy

Currently, the decision to start RRT is based most often on clinical features of volume overload and serum biochemical abnormalities (azotemia, hyperkalemia, severe metabolic acidosis). This overall approach should be based on patient’s clinical context and be individualized. Metabolic acidosis associated with AKI can usually be corrected with bicarbonate and should rarely require urgent dialysis if not accompanied by volume overload or uremic syndrome [73].

There have been no systematic studies showing a definitive advantage for any RRT modality—continuous vs. intermittent RRT—on short-term patient or kidney survival [74]. However, the current consensus is considered continuous RRT to be appropriate for patients with AKI with hemodynamic instability, fluid overload, catabolism, or sepsis. Continuous RRT is also indicated in any patient who meets the criteria for intermittent hemodialysis but cannot undergo this procedure because of hemodynamic instability.

The timing of RRT, early vs. late, after AKI has been studied in observational and prospective studies [75, 76]. Overall, late RRT was associated with a longer duration of RRT and hospital stay and with greater dialysis dependence [77, 78]. The most recent study on this subject is a randomized controlled trial in 231 critically ill patients with AKI (KDIGO stage 2: with ≥ 2-fold increase in serum creatinine from baseline or urinary output < 0.5 mL/kg/h for ≥ 12 h) and plasma NGAL level higher than 150 ng/mL. Early initiation of RRT within 8 h of KDIGO stage 2 AKI significantly reduced 90-day mortality (39.3%) compared with delayed initiation of RRT (54.7%; hazard ratio, 0.66 [95%CI, 0.45 to 0.97], *P* = .03). In the early RRT group, renal function recovery was faster with shorter ICU stay compared with the delayed RRT group [79]. However, this study had limited generalizability as almost all patients recruited were surgical patients and was a single center trial. It should be noted that the conventional indications for RRT in AKI (volume overload, hyperkalemia, severe metabolic acidosis, or uremic symptoms) in this study were not necessarily part of inclusion criteria. In those studies that classic indications were used to compare early vs. delayed RRT, no significant difference in mortality was found [80, 81]. More recent studies that included meta-analysis and randomized multicenter trial showed no added benefit of early initiation of RRT for patients with AKI with respect to dialysis dependence, recovery of renal functions, hospital stay, or all-cause mortality [82, 83]. A strategy of delayed initiation of RRT in critically ill patients with severe AKI obviated the need for RRT in almost 50% of cases [83]. Although there was no mortality difference between early and delayed initiation of RRT groups, the recovery of renal function, as marked by diuresis, was more rapid and catheter-related infections occurred less frequently in the delayed-strategy group than in the early strategy group [83]. In this AKIKI Study Group multicenter trial, the patient had more severe AKI with KDIGO stage 3 reducing the generalizability of the study among different staging.

Evidence from large clinical trials on RRT suggests that the mean duration of treatment is 12–13 days. In clinical practice, daily assessment of both the intrinsic kidney function and the ongoing appropriateness of RRT are required. The recovery of native kidney function can be assessed during RRT by the serial measurement of serum creatinine as well as attention to urine output. Solute clearance of 25–35 mL/min during RRT will result in a stable serum creatinine after 48 h, and further reduction may imply some return of native function. As the renal tubular cells regenerate and re-establish a normal tubular membrane, glomerular filtration will resume and urine output will increase. Increased spontaneous urinary output > 400 mL/day has been described as a good predictor of successful discontinuation of RRT. Conversely, CKD was a strong negative predictor of successful discontinuation of RRT [84].

## Conclusion

AKI is prevalent in critically ill patients in ICU. The most common etiologies of AKI in these patients are due to fluid volume deficit or kidney hypoperfusion and ATN due to shock, inflammatory state, or nephrotoxic drugs. Early recognition of pathophysiology of AKI by careful review of patient’s history and hospital course and intravascular volume assessment complemented by urine biochemical analysis and urine microscopy should guide management strategy in order to reduce further progression of AKI and mortality.

## Abbreviations

ACE: Angiotensin converting enzyme
AKI: Acute kidney injury
ATN: Acute tubular necrosis
CKD: Chronic kidney disease
CVP: Central venous pressure
GFR: Glomerular filtration rate
ICU: Intensive care unit
IGFBP3: Insulin-like growth factor-binding protein 3
KDIGO: Kidney Disease: Improving Global Outcomes
KIM-1: Kidney injury molecule-1
NGAL: Neutrophil gelatinase-associated lipocalin
NSAIDs: Non-steroidal anti-inflammatory drugs
PPV: Positive predictive value
RIFLE criteria: Risk, Injury, Failure, Loss, End Stage
RRT: Renal replacement therapy
TIMP2: Tissue inhibitor of metalloproteinases 2
